# A calibrated deep learning ensemble for abnormality detection in musculoskeletal radiographs

**DOI:** 10.1038/s41598-021-88578-w

**Published:** 2021-04-27

**Authors:** Minliang He, Xuming Wang, Yijun Zhao

**Affiliations:** 1 Gabelli School of Business, Fordham University, New York, NY 10023 USA; 2 Computer and Information Science Department, Fordham University, 113 W 60th St., New York, NY 10023 USA

**Keywords:** Bone imaging, Machine learning, Image processing, Bone

## Abstract

Musculoskeletal disorders affect the locomotor system and are the leading contributor to disability worldwide. Patients suffer chronic pain and limitations in mobility, dexterity, and functional ability. Musculoskeletal (bone) X-ray is an essential tool in diagnosing the abnormalities. In recent years, deep learning algorithms have increasingly been applied in musculoskeletal radiology and have produced remarkable results. In our study, we introduce a new calibrated ensemble of deep learners for the task of identifying abnormal musculoskeletal radiographs. Our model leverages the strengths of three baseline deep neural networks (ConvNet, ResNet, and DenseNet), which are typically employed either directly or as the backbone architecture in the existing deep learning-based approaches in this domain. Experimental results based on the public MURA dataset demonstrate that our proposed model outperforms three individual models and a traditional ensemble learner, achieving an overall performance of (AUC: 0.93, Accuracy: 0.87, Precision: 0.93, Recall: 0.81, Cohen’s kappa: 0.74). The model also outperforms expert radiologists in three out of the seven upper extremity anatomical regions with a leading performance of (AUC: 0.97, Accuracy: 0.93, Precision: 0.90, Recall:0.97, Cohen’s kappa: 0.85) in the humerus region. We further apply the class activation map technique to highlight the areas essential to our model’s decision-making process. Given that the best radiologist performance is between 0.73 and 0.78 in Cohen’s kappa statistic, our study provides convincing results supporting the utility of a calibrated ensemble approach for assessing abnormalities in musculoskeletal X-rays.

## Introduction

Musculoskeletal disorders are injuries or pain in the human musculoskeletal system, including the joints, ligaments, muscles, nerves, tendons, and structures that support the limbs, neck, and back. They are the second leading cause of disability^1^, affecting more than 1.7 billion people worldwide and responsible for 30 million emergency department visits annually. Musculoskeletal conditions significantly limit mobility and dexterity, leading to early retirement from work, reduced accumulated wealth and reduced ability to participate in social roles. While the prevalence of musculoskeletal conditions increases with age, younger people are also affected, often during their peak income-earning years.

A musculoskeletal (bone) X-ray provides practitioners with clear images of the human skeletal system to help determine degenerative damage or injury in the bones and joints of the body. Although X-ray is widely used to assess bone injuries and joint abnormalities, confidence in reading musculoskeletal X-rays comes from years of experience and knowledge that requires extensive training. The best radiologist performance is between 0.73 to 0.78 in Cohen’s kappa statistic^2^.

In recent years, deep learning^3^ (DL) has attracted a great amount of interest and has become a rapidly emerging field in artificial intelligence. In medical image analysis, deep neural networks have been extensively applied to radiology images, including X-rays, B-scans, and MRIs, to help provide even greater diagnostic and treatment capabilities. Facilitated by large datasets and powerful machines, deep learning models have achieved exceptional performance on various tasks such as detecting breast cancer on mammograms^4^, brain tumor segmentation^5^ and classifying interstitial lung disease with high-resolution chest CT Scans^6^. In the musculoskeletal radiology domain, researchers have introduced many successful deep learning approaches, which we divide into categories based on the backbone architecture employed. Arguably, the most popular choices of the underlying model structure are vanilla convolutional neural networks (ConvNet)^7^, residual neural networks (ResNet)^8^, and dense convolutional networks (DenseNet)^9^.

In this study, we present a new calibrated ensemble approach based on the aforementioned three deep neural networks for the task of detecting musculoskeletal abnormalities. Our model was motivated by the individualized proficiencies the three models displayed during the model training phase. Specifically, the three baseline models tended to have similar predicative power on overall studies (i.e., scans of all body parts), however, their efficacies differed significantly depending on the target anatomical region. In practice, such information could be overlooked when the overarching goal is high overall performance. To capitalize on the advantages of each individual model, we propose a calibrated ensemble approach trained using different deep architectures for different body parts. We compare the performance of our model to that of the three baseline models and a traditional meta-learner consisting of ResNet+DenseNet. Experimental results based on the MURA dataset^2^ demonstrate that our proposed model consistently outperforms all four models across five performance evaluation metrics. The model also outperforms expert radiologists in three out of the seven upper extremity anatomical regions.

Model interpretability has always been a limiting factor for DL-based approaches. In the medical domain, the transparency of a model’s decision making process is critical in validating the model, and is often a prerequisite before its clinical deployment. To facilitate our model’s interpretability, we resorted to the class activation map (CAM)^10^, a technique which can be used to visualize the regions used by the model in making its predictions. In the "Class activation map (CAM)" section, we illustrate effective localizations of abnormal musculoskeletal regions using CAMs.

## Related work

Applying machine learning techniques to identify musculoskeletal disorders is an active area of research^11–14^. In earlier studies, Cao et al. presented a generalized bone fracture detection method using features extracted from bone X-ray images and a novel discriminative learning framework called the Stacked Random Forests Feature Fusion^15^. Their method is able to capture 81.2% of the fracture findings reported by radiologists. Boissoneault et al.^16^ investigated the correlations between brain abnormalities and chronic musculoskeletal pain conditions that can be used for illness classification. The authors applied machine learning techniques to separate brain MRI-based biomarkers of chronic pain patients from healthy controls with high accuracy (70–92%).

Recent advances in computer vision and the increased availability of large radiological datasets have enabled deep learning models to achieve performance comparable to medical professionals in a wide variety of musculoskeletal diagnosis tasks. Existing DL-based approaches can be further classified into three categories depending on the type of backbone neural network adopted in the model architecture. The first category consists of approaches based on vanilla convolutional neural networks (ConvNet)^17–22^. ConvNet is arguably the most popular underlying structure adopted by researchers, attributing to the highly successful VGG^7^ model best known for its proficiency in extracting image features. The other two categories are ResNet-based^23–25^ and DenseNet-based approaches^2,23,26^.

It is worth noting that most of the existing studies are extensions of the three aforementioned base deep learning structures. For example, Saif et al. proposed a capsule network addressing the limitations of ConvNet when aggregating large amounts of data is not possible^21^. Although some of these studies compared the performance of models with different backbone networks, the studies are mostly limited to one or a few particular types of anatomical regions. For example, Mondol et al. compared the performance of ConvNet, ResNet, and some ensemble methods on four types (wrist, humerus, finger and elbow) of studies^25^. Tiulpin et al. applied a deep Siamese ConvNet to automatically score knee osteoarthritis severity according to the Kellgren-Lawrence grading scale^22^. In our study, we evaluate the performance of these three backbone deep neural networks on overall and seven standard upper extremity regions.

In addition to designing individual deep learning models, researchers have also explored the ensemble technique^27^ in search of more robust and superior performance^25,28–30^. In a recent study, Jones et al. introduced an effective deep-learning system to detect fractures across the musculoskeletal system using an ensemble of ten ConvNets^28^. Compared to Jones et al.’s study, our research bears a similar mission but is different in three aspects. First, our work is more comprehensive and challenging because the types of disorder in our study are not limited to fractures. The additional abnormalities present in our dataset include hardware, joint diseases, lesions, and subluxations^2^. Second, our model is a heterogeneous ensemble of different deep networks, whereas their system is a homogeneous ensemble of the same type of neural network, i.e., ConvNets. Last, we propose a new ensemble methodology that employs different learners for different body parts, whereas their algorithm is a traditional ensemble in which each learner is trained using the entire dataset. Performance-wise, depending on anatomical regions, Jones et al. report AUC scores ranging from 0.888 to 0.98. Our approach achieves comparable efficacy with AUC scores ranging from 0.87 to 0.97 in a more comprehensive study. We introduce our new calibrated ensemble learner in the "Methods" section.

## Contributions

The main contribution of this work is a new ensemble learning approach that capitalizes the strength of individual learners in building predictive models. In the clinical setting, the resulting models can be interpreted as experts, each of them specializes in a particular type of patient or abnormality. Our method can be applied to any dataset with distinct data subgroups.

Another contribution of our work is presenting a use case of the CAM technique to alleviates the “black box” limitation of neural network models. Our findings demonstrate the effectiveness of the CAM, which helps to build trust towards adopting DL-based automatic methods in clinical practices.

## Data and preprocessing

### Dataset

**Figure 1:**
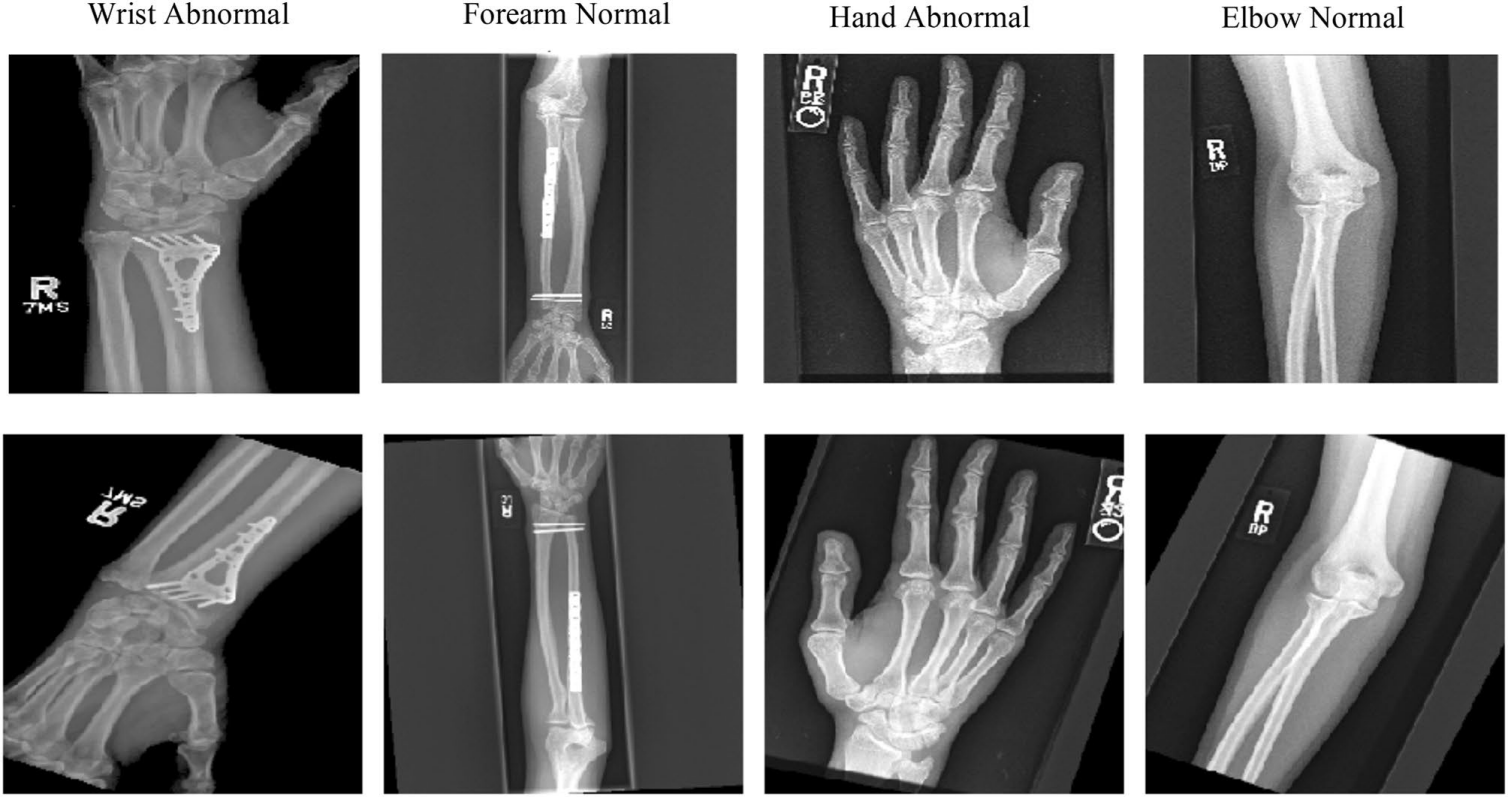
Samples training images. The first row are original images from the MURA dataset. The second row presents images augmented in three sequential operations: a random horizontal flip, a random vertical flip, and a random rotation within $-\,30^\circ$ to $30^\circ$.

For our models’ training and evaluation, we used the MURA dataset^2^ provided by the Stanford ML Group. Our experimental data consists of 14,656 studies from 11,967 patients, with a total of 40,005 publicly available multi-view radiographic images. Each image belongs to one of the seven standard upper extremity studies: *Elbow, Finger, Forearm, Hand, Humerus, Shoulder*, and *Wrist*. Table 1 presents the statistics of the number of positive (abnormal) and negative (normal) images in each type of study. Each image was manually labeled as normal or abnormal by board-certified radiologists from the Stanford Hospital at the time of the clinical acquisition of the radiography^2^. The first row in Fig. 1 presents sample normal and abnormal images in the MURA dataset. 

In addition, the types of abnormalities present in the dataset were studied by reviewing the radiologist reports and manually labeling 100 abnormal images, with the following finding: 53 studies were labeled with fractures, 48 with hardware, 35 with degenerative joint diseases, and 29 with other miscellaneous abnormalities, including lesions and subluxations^2^.

**Table 1:** Number of images in each anatomical category.

	Train		Validation		Test		Total	
	Positive*	Negative*	Positive	Negative	Positive	Negative	Positive	Negative
Elbow	1734	2584	272	341	230	235	2236	3160
Finger	1710	2750	258	388	247	214	2215	3352
Forearm	583	1042	78	122	151	150	812	1314
Hand	1287	3549	197	510	189	271	1673	4330
Humerus	514	593	85	80	140	148	739	812
Shoulder	3627	3673	541	538	278	285	4446	4496
Wrist	3489	4993	498	772	295	364	4282	6129
Total	12,944	19,184	1929	2751	1530	1667	16,403	23,602
	32,128		4680		3197		40,005	

*Positive or negative represents abnormal or normal, respectively.

### Data preprocessing

The images in the MURA dataset are of different sizes. The width ranges from 89 to 512, and the height ranges from 132 to 512. We resized them to standard 256 by 256 grayscale images to facilitate the training of our models. The input size was selected experimentally to preserve as much information as possible and simultaneously compensate for the large computational cost during the model training. We then applied standard min-max normalization^31^ to scale the pixel values from [0, 255] to [0, 1].

We applied three image augmentation techniques to boost the performance of our deep networks: horizontal flip, vertical flip, and rotation between $-30^\circ$ and $30^\circ$. A random sequence of one to three of these transformations was applied to each image in the training data. We experimented with other augmentation methods, including random brightness change, which turned out to be less effective. The second row in Fig. 1 presents samples of randomly augmented input images corresponding to the original images in the first row.

## Results

We trained our deep learning models using the Adam optimizer with the hyper-parameters set experimentally at 0.9 for the exponential decay rate for the first moment estimates (i.e., $\beta _1$), 0.999 for the exponential decay rate for the second moment estimates ($\beta _2$), and 0.001 for the learning rate (varying learning rates resulted in similar performance).

The large size of our training data (Table 1) makes it challenging to load the entire dataset in memory even with the most state-of-the-art system configuration. To this end, we employed a data generator^32^ to dynamically generate the training batches and feed them to the model training procedures. The batch size was set to 32, except for DenseNet for which the batch size was set to 16 due to its large memory consumption during training.

We found it was sufficient to set the training epochs at 100 with an early stopping condition monitoring the convergence of the training loss. With the help of the *ModelCheckpoint* callback function from the Keras package, the best model was selected as the one with the highest validation set performance among all training epochs. Table 2 presents the total number of parameters for each model and the training time.

**Table 2:** Model training statistics.

	Parameters	Batch size	Epochs	Training time** (h)
ConvNet	18,894,578	32	100	12
ResNet	21,170,050	32		24
DenseNet	12,639,938	16		60

**Using Intel(R) Core(TM) i7-8750H CPU processor with 16GB Ram and NVIDIA GeForce GTX 1070 with Max-Q Design GPU.

### Evaluation metrics

We evaluated the performance of our models using five metrics defined as follows:

- *AUC score* (of the ROC curve): A ROC curve^33^ displays the trade-off between the True Positive Rate (TPR, or *Sensitivity*) and the True Negative Rate (TNR, or *Specificity*) of a classification model at different threshold settings. AUC reveals the capability of a model to separate the positive and negative classes, i.e., the higher the AUC score, the more effective a model is at performing the classification.
- *Accuracy*: the fraction of correctly classified images in the test data.
- *Precision*: the fraction of correctly classified images among all positive (abnormal) predictions.
- *Recall*: the fraction of correctly classified images among all positive (abnormal) images in the test data.
- * Cohen’s kappa coefficient* ($\kappa$): measures inter-rater reliability for categorical items^34^. It is considered to be a more robust measure than a simple percent agreement calculation.

### Performance comparison and analysis

Because we applied image augmentation techniques ("Data preprocessing" section) to boost our models’ performance, each X-ray study contains a collection of multi-view images from different angles. We define the abnormality probability of an X-ray study to be the average predicted probabilities for the positive class of all images in the study. Final classification decisions are based on a threshold of 0.5 on the average predicted probabilities.

**Figure 2:**
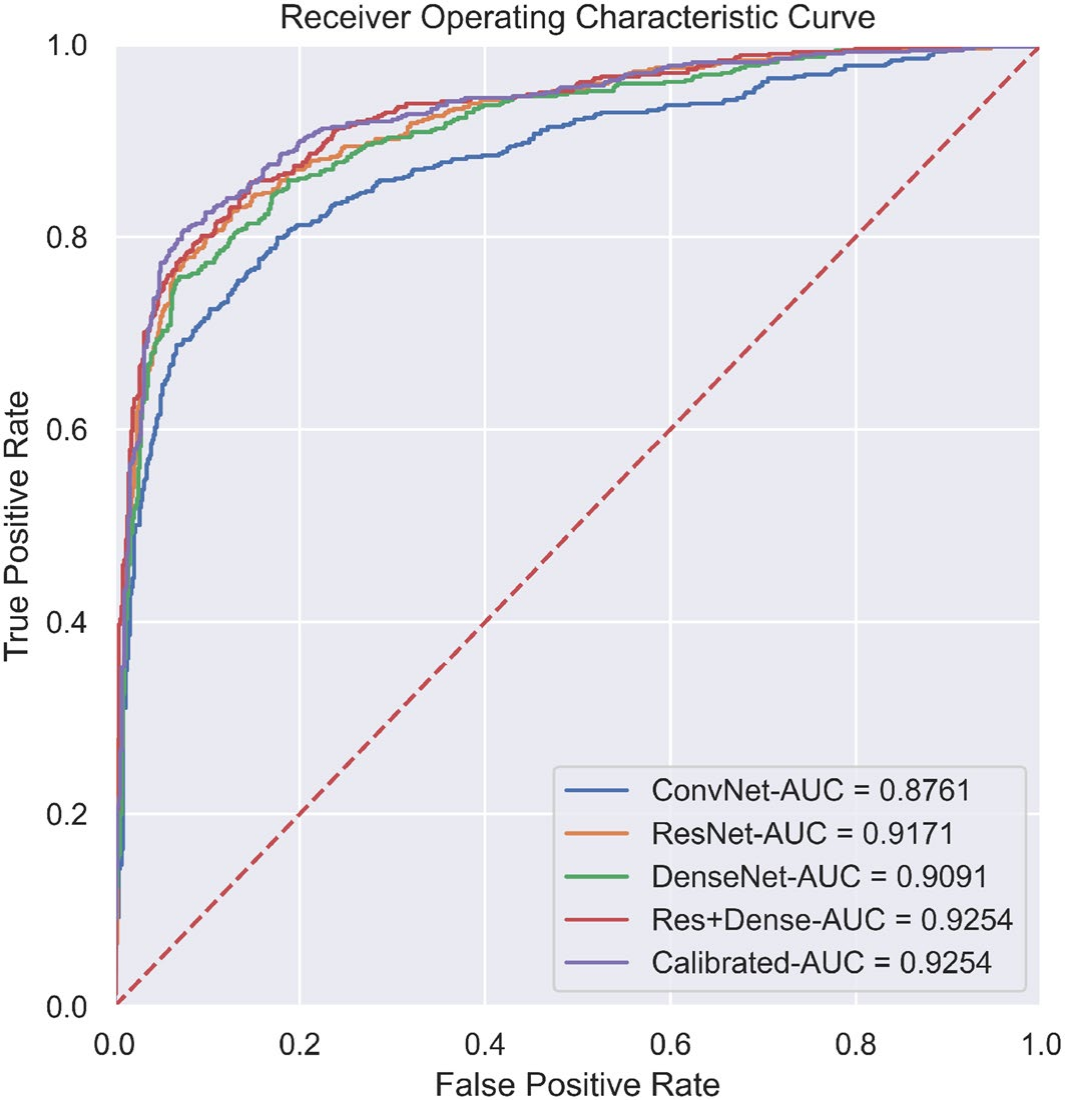
ROC curves of five models.

Figure 2 presents the AUC scores and the corresponding ROC curves of the five models outlined in the "Methods" section for the overall study. We observe that, except for ConvNet, the other four models have similar proficiencies in detecting abnormal scans. Furthermore, the two ensemble learners hold a marginal advantage over the individual models.

Table 3 presents the performance comparison between our five models on overall and individual studies. We group the comparisons by the five performance metrics (Column 1) described in the "Evaluation metrics" section. Each group displays its models’ overall and study-type specific performance on the test dataset. Using ConvNet as our baseline model, the numbers in parentheses indicate the percentage change ($\Delta$) in a particular evaluation metric for model *M* over the baseline. Specifically, $\Delta =(P_M - P_{ConvNet})/P_{ConvNet}$, where $P_M$ and $P_{ConvNet}$ are performance scores of model *M* and ConvNet, respectively. We first examine the overall performance (Column 3). The results are summarized as follows:

**Table 3:** Performance comparison of five models—overall and across different parts of body.

Metric	Model	Overall	Elbow	Finger	Forearm	Hand	Humerus	Shoulder	Wrist
AUC	ConvNet	0.88	0.89	0.87	0.88	0.84	0.90	0.83	0.93
	ResNet	0.92 (5%)	0.89	0.92	0.89	0.85	**0.97**	**0.90**	**0.97**
	DenseNet	0.91 (4%)	**0.91**	0.91	0.90	0.88	0.94	0.86	0.95
	Res+Dense	**0.93 (6%)**	**0.91**	**0.93**	0.90	**0.89**	**0.97**	**0.90**	**0.97**
	Calibrated	**0.93 (6%)**	**0.91**	**0.93**	**0.91**	0.87	**0.97**	**0.90**	**0.97**
Accuracy	ConvNet	0.82	0.87	0.80	0.80	0.78	0.84	0.77	0.89
	ResNet	0.86 (4%)	0.83	0.85	0.83	0.81	0.92	**0.85**	0.90
	DenseNet	0.85 (4%)	**0.90**	0.83	0.84	0.81	0.87	0.81	0.89
	Res+Dense	0.86 (5%)	0.89	0.84	0.83	0.81	0.90	0.83	**0.92**
	Calibrated	**0.87 (6%)**	**0.90**	**0.86**	**0.86**	**0.87**	**0.93**	**0.85**	**0.92**
Precision	ConvNet	0.86	0.83	0.87	0.85	0.89	0.88	0.78	0.92
	ResNet	0.91 (5%)	0.93	0.89	0.90	0.92	**0.90**	**0.86**	0.94
	DenseNet	0.90 (4%)	0.93	0.89	0.90	0.91	0.88	0.84	0.92
	Res+Dense	0.91 (6%)	0.95	0.90	0.95	0.89	0.89	0.85	0.95
	Calibrated	**0.93 (8%)**	**0.96**	**0.93**	**0.96**	**0.93**	**0.90**	**0.86**	**0.96**
Recall	ConvNet	0.72	0.71	0.72	0.71	0.60	0.78	0.75	0.75
	ResNet	0.78 (8%)	0.74	0.75	0.72	0.61	0.94	0.83	0.81
	DenseNet	0.76 (6%)	0.82	0.75	0.70	0.59	0.87	0.76	0.80
	Res+Dense	0.77 (8%)	0.79	0.75	0.72	0.56	0.93	0.81	0.82
	Calibrated	**0.81 (12%)**	**0.82**	**0.94**	**0.80**	**0.75**	**0.97**	**0.83**	**0.87**
Cohen’s kappa ($\kappa$)	ConvNet	0.63	0.72	0.59	0.59	0.51	0.67	0.55	0.76
	ResNet	0.71 (12%)	0.64	0.70	0.65	0.59	0.84	**0.70**	0.80
	DenseNet	0.70 (10%)	**0.79**	0.67	0.68	0.59	0.75	0.63	0.77
	Res+Dense	0.72 (13%)	0.77	0.68	0.65	0.57	0.81	0.66	**0.82**
	Calibrated	**0.74 (17%)**	**0.79**	**0.73**	**0.71**	**0.72**	**0.85**	**0.70**	**0.82**

Bold numbers indicate the highest performing model(s) for each evaluation metric across eight studies. The numbers in parenthesis indicate the percentage change $(P_M - P_{ConvNet})/P_{ConvNet}$ in a particular evaluation metric for a model M over the baseline ConvNet model. “Res+Dense” is a meta-learner which makes its predictions based on the average of the output probabilities from the ResNet and DenseNet models. “Calibrated” is our proposed ensemble model which trains a designated deep learner for each anatomical region.

- The ResNet model outperforms ConvNet in all five measures with 5%, 4%, 5%, 8%, and 12% improvement in AUC, Accuracy, Precision, Recall, and $\kappa$ respectively.
- The DenseNet model outperforms ConvNet in all five measures with 4%, 4%, 4%, 6%, and 10% improvement in AUC, Accuracy, Precision, Recall, and $\kappa$ respectively.
- The Res+Dense model outperforms ConvNet in all five measures with 6%, 5%, 6%, 8%, and 13% improvement in AUC, Accuracy, Precision, Recall, and $\kappa$ respectively.
- The Calibrated model outperforms ConvNet in all five measures with 6%, 6%, 8%, 12%, and 17% improvement in AUC, Accuracy, Precision, Recall, and $\kappa$ respectively.

From Column 4–10 of Table 3, we can examine the models’ performance on images of various body parts. The results are summarized as follows:

- The last four models consistently outperform the baseline ConvNet across all evaluation metrics and specific body part studies.
- Among the three individual models, ResNet is the best at detecting abnormalities in the *Humerus* (AUC: 0.97, Accuracy: 0.92, Precision: 0.90, Recall: 0.94, $\kappa$: 0.84), *Shoulder* (AUC:0.90, Accuracy: 0.85, Precision: 0.86, Recall: 0.83, $\kappa$: 0.70), and *Finger* (AUC:0.92, Accuracy: 0.85, Precision: 0.89, Recall: 0.75, $\kappa$: 0.70 ) regions.
- Among the three individual models, DenseNet is the best at detecting injuries in the *Elbow* region (AUC: 0.91, Accuracy: 0.90, Precision: 0.93, Recall: 0.82, $\kappa$: 0.79).
- ResNet and DenseNet have comparable performance at identifying abnormalities in *Forearm*, *Hand* and *Wrist* studies. However, the first two categories have significantly lower $\kappa$ values compared to the last one. Both models excel at *Wrist* studies with (AUC: 0.97, Accuracy: 0.9, Precision: 0.94, Recall: 0.81,$\kappa$: 0.80) and (AUC: 0.95, Accuracy: 0.89, Precision: 0.92, Recall: 0.80, $\kappa$: 0.77), respectively.
- Res+Dense achieves comparable or superior AUC scores compared to those from the two individual models across all categories, suggesting that the ensemble model has more discriminative power and would be a more desirable model compared to individual learners.
- Our “Calibrated” model displays the highest performance across all categories and measures. For some studies (e.g., *Hand*, *Forearm*, and *Finger*), we observe significantly improved $\kappa$ values over the other models.
- Our “Calibrated” model achieved $\kappa$ values above 0.7 across all eight studies. Given that the best radiologist performance is between 73 and 78% depending on the parts of the body imaged^2^, our “Calibrated” model exhibits performance superior to human experts in *Elbow*, *Humerus* and *Wrist* studies.

### Class activation map (CAM)

The Class Activation Map (CAM) is a technique that explicitly enables neural networks to have localization ability despite being trained on image level labels^10^. A CAM for a particular class indicates the discriminative region(s) used by the model to make the prediction. Thus, CAMs serve as another method to evaluate the efficacy of convolutional-based models.

**Figure 3:**
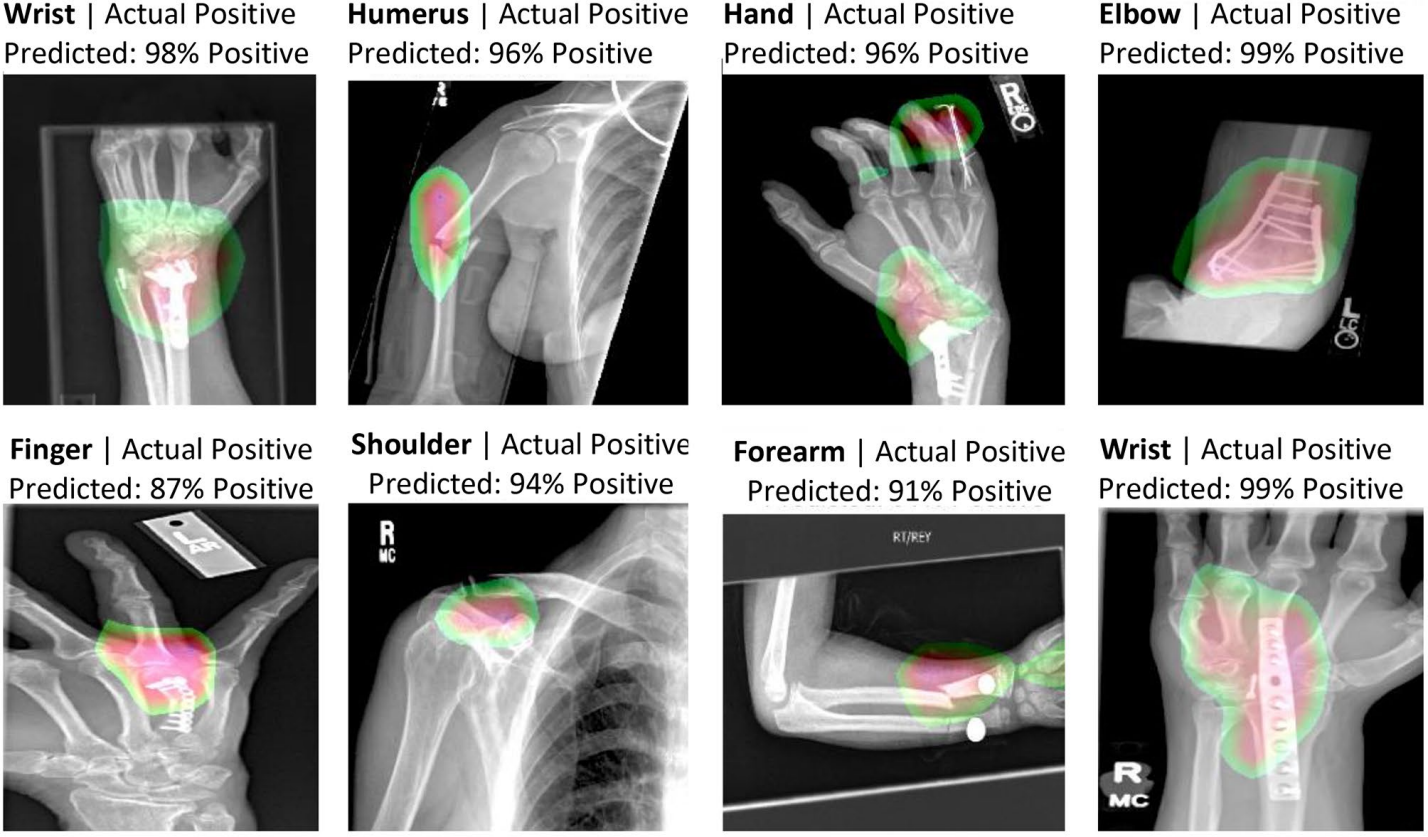
Sample class activation maps.

By extracting the final Conv2D layer in our best performing calibrated model and mapping the corresponding output on each original image, we can highlight the region(s) of an image and visualize our model’s attention while making its prediction. Figure 3 presents the CAM regions identified by our model on sample images with high confidence positive scores. We observe that our model correctly pinpointed the problematic regions in all cases with high confidence in its positive prediction.

## Discussion

In this study, we evaluated five deep learning approaches for the task of detecting abnormalities using musculoskeletal radiographs. Our experimental results suggest that ResNet and DenseNet have similar advantages over ConvNet in the overall study, however, their performance on different anatomical regions varied significantly. Furthermore, both ensemble approaches are more effective compared to the individual models. The calibrated ensemble approach consistently outperforms the other four models across all five evaluation metrics. For three out of seven specific anatomical regions (i.e., *Elbow*, *Humerus*, and *Wrist*), the calibrated model achieved performance superior to expert radiologists.

Two factors could have contributed to the success of our CE algorithm. The first one is the implicit i.i.d. assumption made by all machine learning algorithms, i.e., training and test samples are drawn independently from an identical distribution. This assumption is better enforced when we restrict our data to images from specific anatomical regions, thereby allowing greater potential for success. The second factor is that different deep neural networks can be most effective for different regions due to their customized architectures. The CE algorithm exploited the individual strength of each learner by observing their behavior in the validation data. Our proposed approach can be extended to other applications where unique subgroups are present in the data. Unlike traditional ensemble approaches in which each learner is trained using the entire dataset, the calibrated ensemble would designate a desired learner for each idiosyncratic data component.

One limitation of our approach is the requirement of a pre-defined partition of data subgroups. In our study, there is an unambiguous effective division of data using anatomical regions. In practice, not all datasets exhibit straightforward distinctive data clusters. For future work, we foresee a potential integration with unsupervised clustering algorithms to help discover the underlying subgroups of the data with similar characteristics.

Lastly, we applied the CAM technique to highlight the region(s) essential to the network's decision making process. Such information alleviates the “black box” limitation of DL-based approaches and makes a model’s decision process transparent to practitioners. Our findings demonstrate the effectiveness of the CAM, which helps to build trust towards adopting DL-based automatic methods in clinical practices.

## Methods

In this section we introduce the five deep learning methods we employed to conduct our experiments. The first three (ConvNet, ResNet, and DenseNet) are models that have been widely adopted by researchers. The other two are meta-learner Res+Dense and our proposed calibrated ensemble learner. We elaborate our method of addressing the data imbalance issue in the “Customized loss function" section.

### Convolutional neural network (ConvNet)

ConvNet is a class of deep neural networks designed to analyze visual images. A ConvNet model consists of multiple convolutional layers which serve the roles of extracting image features, ranging from simple patterns (e.g., edges, curves, etc.) to complex figures (e.g., hands, elbows, shoulders, etc.). The subsampling (e.g., maxpooling) layers control the intermediate feature map sizes between the consecutive convolutions. While the convolutional layers act as feature detectors, the fully connected (FC) layers perform classification based on the features extracted by the convolutional layers.

Table 4 illustrates the architecture of our ConvNet model. Our model is a modified version of the popular VGGNet [2] architecture. We adjusted the sizes of some convolutional and FC layers in the original VGG model. These customizations were motivated by the large discrepancy between the training and test performance based on the VGG-16 model, which indicated potential overfitting. We further applied batch normalization (BN) after each convolutional layer.

**Table 4:** ConvNet architecture.

	Filter Size	Stride	# of Filters
Convolutional layer 1	3 × 3	(1,1)	16
Convolutional layer 2	3 × 3	(1,1)	16
Max pooling	2 × 2		
Convolutional layer 3	3 × 3	(1,1)	32
Convolutional layer 4	3 × 3	(1,1)	32
Max pooling	2 × 2		
Convolutional layer 5	3 × 3	(1,1)	64
Convolutional layer 6	3 × 3	(1,1)	64
Max pooling	2 × 2		
Convolutional layer 7	3 × 3	(1,1)	128
Convolutional layer 8	3 × 3	(1,1)	128
Max pooling	2 × 2		
Convolutional layer 9	3 × 3	(1,1)	256
Convolutional layer 10	3 × 3	(1,1)	256
Max pooling	2 × 2		
Convolutional layer 11	3 × 3	(1,1)	512
Convolutional layer 12	3 × 3	(1,1)	512
Max pooling	2 × 2		
Convolutional layer 13	3 × 3	(1,1)	1024
Convolutional layer 14	3 × 3	(1,1)	1024
Max pooling	2 × 2		
	Flatten		
FC 1	512		
FC 2	256		

### Residual neural network (ResNet)

ResNet^35^ attracted a great amount of attention after it won all five main tracks of ILSVRC^36^ and CoCo^37^ image detection, localization, and segmentation competitions in 2015. In the ILSVRC classification competition, ResNet achieved 3.6% top 5 error (i.e., correct answer not present among top 5 predictions), a performance comparable, if not superior, to human ability.

**Figure 4:**
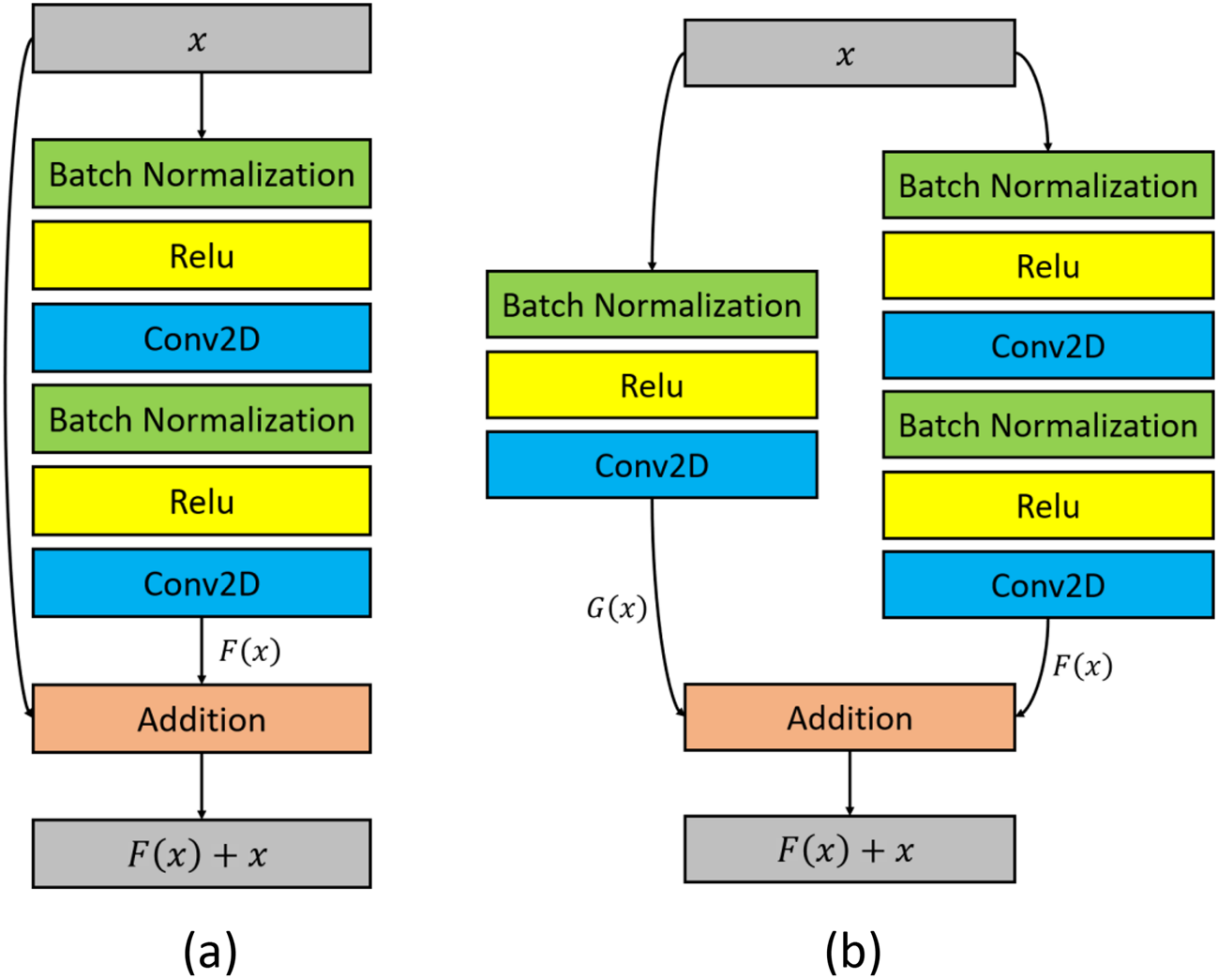
Two types of residual blocks.

The most noticeable characteristic of ResNet is its “ultra deep” architecture. Due to the vanishing/exploding gradients problem^38^, a deep learning model is often limited in the total number of convolution layers. ResNet addresses this issue by introducing the “ResNet block” with skip-connections to feed the activation function directly into layers much deeper into the model. As illustrated in Fig. 4a, instead of learning $y = M(x)$, ResNet is designed to learn the residual function *F*(*x*), where $M(x) = F(x) + x$. Figure 4b presents another type of “ResNet block”^8^ capable of accommodating for the desired number of feature maps in each layer. Furthermore, blocks in Fig. 4 adopt the “full pre-activation” design illustrated by He et al.^8^, in which batch normalization (BN) and activation (ReLU) proceed each convolution (Conv2D) layer. Our ResNet consists of a mixture of 25 convolutional blocks of both types.

### Dense convolutional network (DenseNet)

DenseNet was first introduced in 2017^9^. It extended the idea of skip-connections in ResNet to dense connections. In particular, for each layer, the feature maps of all preceding layers are used as inputs, and its own feature maps are fed into all subsequent layers. Since each layer receives feature maps from all preceding layers, DenseNets strengthen feature propagation, encourage feature reuse, and substantially reduce the number of parameters. We trained our DenseNet model using the pre-implemented *DenseNet169* structure provided with the Tensorflow Keras package^39^, which consists of 169 convolutional layers.

### Ensemble of ResNet and DenseNet (Res+Dense)

Ensemble learning has been proven to produce improved and more robust performance than a single model^27^. To further evaluate the performance of our proposed model, we compare its performance to a traditional meta-learner *Res+Dense* which is constructed by combining the outcomes from these two baseline models. Our experimental findings suggest that including ConvNet will not further improve the ensemble model’s performance. The *Res+Dense* learner makes its predictions based on the average of the output probabilistic scores from the ResNet and DenseNet models. Formally,

$$\begin{aligned} {\hat{y}}(x_i) = {\left\{ \begin{array}{ll} 1 &{}\quad \text {if } ( p_r+ p_d) \ge 1\\ 0 &{}\quad \text {otherwise} \end{array}\right. } \end{aligned}$$

where ${\hat{y}}$ is the predicted label for instance $x_i$. $p_r$ and $p_d$ are the predicted abnormality probabilities for instance $x_i$ from the ResNet and DenseNet, respectively. Our meta-learner outperforms standalone classifiers in overall performance across all five evaluation metrics outlined in the "Evaluation metrics" section.

**Table 5:** Validation dataset performance of baseline models—overall and across different parts of body.

Metric	Model	Overall	Elbow	Finger	Forearm	Hand	Humerus	Shoulder	Wrist
AUC	ConvNet	0.89	0.90	0.90	0.85	0.84	0.91	0.84	0.92
	ResNet	0.91	0.91	**0.93**	0.88	**0.86**	**0.96**	**0.89**	**0.96**
	DenseNet	0.91	**0.93**	0.92	**0.90**	0.85	0.95	0.86	0.95
Accuracy	ConvNet	0.84	0.85	0.83	0.84	0.78	0.83	0.79	0.87
	ResNet	0.86	0.84	**0.87**	0.85	**0.82**	**0.90**	**0.84**	**0.91**
	DenseNet	0.85	**0.88**	0.84	**0.86**	**0.82**	0.88	0.82	0.89
Cohen’s kappa ($\kappa$)	ConvNet	0.64	0.73	0.64	0.64	0.52	0.68	0.58	0.77
	ResNet	0.72	0.67	**0.68**	0.67	**0.60**	**0.85**	**0.71**	**0.80**
	DenseNet	0.71	**0.80**	0.65	**0.70**	0.59	0.75	0.63	0.77

Bold numbers indicate the highest performing model for each evaluation metric across eight studies. DenseNet is the designated learner for the *Elbow* and *Forearm* studies and ResNet for the remaining ones.

### Calibrated ensemble model

In training our deep learning models, we found that different classifiers excel at analyzing studies of different parts of the body. Table 5 presents the performance of three individual models on the validation data evaluated using AUC, accuracy, and Cohen’s $\kappa$. We observe that the overall performance of ResNet and DenseNet were highly similar, and both models outperformed the ConvNet. However, if we examine the specific anatomical regions, DenseNet showed a noticeable advantage over the ResNet for the *Elbow* study across all three performance measures, especially in Cohen’s $\kappa$ (ResNet: 0.67; DenseNet: 0.80). Similarly, DenseNet outperformed ResNet for the *Forearm* region. On the other hand, ResNet was more adept in classifying *Humerus* and *Shoulder* images compared to the DenseNet, with Cohen’s $\kappa$ values at (ResNet:0.85; DenseNet: 0.75) and (ResNet: 0.71; DenseNet: 0.63) , respectively.

Leveraging these findings, we trained a customized ensemble model that would designate a deep learner for each specific type of study. Specifically, for each anatomical region, we selected the desired learner with the best validation performance in at least two out of the three metrics as presented in Table 5. As a result, DenseNet was selected for *Elbow* and *Forearm* studies, and ResNet for the remaining categories.

We calibrated our ensemble model by fine-tuning each learner with its corresponding subset of training images. Each model’s weights were initialized using the pre-trained weights of its overall model. Experimental results on the test data (Table 3) demonstrate our calibrated model not only achieves the highest performance on individual studies but also leads the performance in the “Overall” category. Furthermore, the model’s advantage is consistent across all five evaluation metrics.

### Customized loss function

We select cross-entropy (CE) as the loss function for training our models. However, from Table 1, we observe that the number of positive and negative instances is imbalanced in the majority of the seven body parts studies. Applying standard machine learning algorithms to an imbalanced dataset often leads to unsatisfactory performance on the minority class, which is the more interesting and important class in our task. To address this issue, we modified the standard CE loss function to be a weighted cross-entropy loss such that the weight for class $i \in \{positive, negative\}$ is inversely proportional to the number of instances in class *i*. For example, when training for a binary classification model for the “*Elbow*” studies, we have 1,734 and 2,584 instances in the positive and negative classes (Table 1), respectively. As a result, the respective weights for the positive and negative classes are set to be 2584/(2584+1734) and 1734/(2584+1734) in the weighted loss function. Formally,

$$\begin{aligned}  \text{Weighted}\_\text{loss} (X,Y)&= - \sum\limits_{x_{i} \in X} \sum _{t \in T} {w_{1}^{t}\cdot {y_i}\ln {p(y_i=1|x_i)}} \\&\quad -\, \sum\limits_{x_{i} \in X} \sum\limits_{t \in T} {w_{0}^{t}\cdot {(1-y_i)}\ln {p(y_i=0|x_i)}}  \end{aligned}$$

where

- $X=\{x_1, x_1, \dots , x_N\}$ are the training instances
- $Y=\{y_1, y_2, \dots y_N \}$ are the ground truth labels for the corresponding instances in *X*
- $p(y_i = v|x_i)$ is the predictive probability for $y_i \in \{0, 1\}$ given instance $x_i$
- $T = \{ Elbow, Finger, Forearm, Hand, Humerus, Shoulder, Wrist\}$ contains the study types
- $|N_t|$ is the number of normal images of study type $t \in T$
- $|A_t|$ is the number of abnormal images of study type $t \in T$
- $w_0^t={|A_t|}/{(|N_t|+|A_t|)}$ is the weight for all normal instances of study type $t \in T$
- $w_1^t=|N_t|/(|N_t|+|A_t|)$ is the weight for all abnormal instances of study type $t \in T.$

## Data Availability

This study is based on the publicly available MURA dataset provided by the Stanford ML Group (reference^2^).

## References

[1] Barbe MF (2013). The interaction of force and repetition on musculoskeletal and neural tissue responses and sensorimotor behavior in a rat model of work-related musculoskeletal disorders. BMC Musculoskelet. Disord..

[2] Rajpurkar, P. *et al.* Mura: large dataset for abnormality detection in musculoskeletal radiographs. arXiv preprint arXiv:1712.06957 (2017).

[3] LeCun Y, Bengio Y, Hinton G (2015). Deep learning. Nature.

[4] Becker AS (2017). Deep learning in mammography: diagnostic accuracy of a multipurpose image analysis software in the detection of breast cancer. Investig. Radiol..

[5] Havaei M (2017). Brain tumor segmentation with deep neural networks. Med. Image Anal..

[6] Chartrand G (2017). Deep learning: a primer for radiologists. Radiographics.

[7] Simonyan, K. & Zisserman, A. Very deep convolutional networks for large-scale image recognition. arXiv preprint arXiv:1409.1556 (2014).

[8] He, K., Zhang, X., Ren, S. & Sun, J. Identity mappings in deep residual networks. In *European Conference on Computer Vision*, 630–645 (Springer, 2016).

[9] Huang, G., Liu, Z., Van Der Maaten, L. & Weinberger, K. Q. Densely connected convolutional networks. In: *Proceedings of the IEEE Conference on Computer Vision and Pattern Recognition* 4700–4708 (2017).

[10] Zhou, B., Khosla, A., Lapedriza, A., Oliva, A. & Torralba, A. Learning deep features for discriminative localization. In: *Proceedings of the IEEE Conference on Computer Vision and Pattern Recognition* 2921–2929 (2016).

[11] Hirschmann, A. *et al.* Artificial intelligence in musculoskeletal imaging: review of current literature, challenges, and trends. In: *Seminars in Musculoskeletal Radiology*, vol. 23, 304–311 (Thieme Medical Publishers, 2019). 10.1055/s-0039-1684024 31163504

[12] Aal, M. M. A. *et al.* Survey: automatic recognition of musculoskeletal disorders from radiographs. In *2018 13th International Conference on Computer Engineering and Systems (ICCES)*, 56–62 (IEEE, 2018).

[13] Liu F, Kijowski R (2019). Deep learning in musculoskeletal imaging. Adv. Clin. Radiol..

[14] Chea P, Mandell JC (2020). Current applications and future directions of deep learning in musculoskeletal radiology. Skelet. Radiol..

[15] Cao, Y., Wang, H., Moradi, M., Prasanna, P. & Syeda-Mahmood, T. F. Fracture detection in x-ray images through stacked random forests feature fusion. In: *2015 IEEE 12th International Symposium on Biomedical Imaging (ISBI)*, 801–805 (IEEE, 2015).

[16] Boissoneault J, Sevel L, Letzen J, Robinson M, Staud R (2017). Biomarkers for musculoskeletal pain conditions: use of brain imaging and machine learning. Curr. Rheumatol. Rep..

[17] Kandel I, Castelli M, Popovič A (2020). Musculoskeletal images classification for detection of fractures using transfer learning. J. Imaging.

[18] Liang S, Gu Y (2020). Towards robust and accurate detection of abnormalities in musculoskeletal radiographs with a multi-network model. Sensors.

[19] Irmakci, I., Anwar, S. M., Torigian, D. A. & Bagci, U. Deep learning for musculoskeletal image analysis. In *2019 53rd Asilomar Conference on Signals, Systems, and Computers*, 1481–1485 (IEEE, 2019).

[20] Goyal M, Malik R, Kumar D, Rathore S, Arora R (2020). Musculoskeletal abnormality detection in medical imaging using GnCNNr (group normalized convolutional neural networks with regularization). SN Comput. Sci..

[21] Saif A, Shahnaz C, Zhu W-P, Ahmad MO (2019). Abnormality detection in musculoskeletal radiographs using capsule network. IEEE Access.

[22] Tiulpin A, Thevenot J, Rahtu E, Lehenkari P, Saarakkala S (2018). Automatic knee osteoarthritis diagnosis from plain radiographs: a deep learning-based approach. Sci. Rep..

[23] Chada G (2019). Machine learning models for abnormality detection in musculoskeletal radiographs. Rep. Med. Cases Images Videos.

[24] Paul HY (2019). Automated semantic labeling of pediatric musculoskeletal radiographs using deep learning. Pediatr. Radiol..

[25] Mondol, T. C., Iqbal, H. & Hashem, M. Deep CNN-based ensemble CADx model for musculoskeletal abnormality detection from radiographs. In *2019 5th International Conference on Advances in Electrical Engineering (ICAEE)*, 392–397 (IEEE, 2019).

[26] Shao, Y. & Wang, X. A two stage method for abnormality diagnosis of musculoskeletal radiographs. In *International Conference on Pattern Recognition and Artificial Intelligence*, 610–621 (Springer, 2020).

[27] Dietterich, T. G. Ensemble methods in machine learning. In *International Workshop on Multiple Classifier Systems*, 1–15 (Springer, 2000).

[28] Jones RM (2020). Assessment of a deep-learning system for fracture detection in musculoskeletal radiographs. NPJ Digit. Med..

[29] Banga, D. & Waiganjo, P. Abnormality detection in musculoskeletal radiographs with convolutional neural networks (ensembles) and performance optimization. arXiv preprint arXiv:1908.02170 (2019).

[30] Paraponaris A, Ba A, Gallic E, Liance Q, Michel P (2019). Predicting musculoskeletal disorders risk using tree-based ensemble methods. Eur. J. Public Heal..

[31] Patro, S. & Sahu, K. K. Normalization: a preprocessing stage. arXiv preprint arXiv:1503.06462 (2015).

[32] Michlin, I. Keras data generators and how to use them (2019).

[33] Fawcett T (2006). An introduction to roc analysis. Pattern Recognit. Lett..

[34] Kraemer, H. C. Kappa coefficient. In *Wiley StatsRef: Statistics Reference Online* 1–4 (2014).

[35] He, K., Zhang, X., Ren, S. & Sun, J. Deep residual learning for image recognition. In: *Proceedings of the IEEE Conference on Computer Vision and Pattern Recognition* 770–778 (2016).

[36] Russakovsky O (2015). Imagenet large scale visual recognition challenge. Int. J. Comput. Vis..

[37] Lin, T.-Y. *et al.* Microsoft coco: common objects in context. In *European conference on computer vision*, 740–755 (Springer, 2014).

[38] Pascanu, R., Mikolov, T. & Bengio, Y. On the difficulty of training recurrent neural networks. In: *International Conference on Machine Learning* 1310–1318, (2013).

[39] Brownlee, J. *Deep Learning with Python: Develop Deep Learning Models on Theano and TensorFlow Using Keras* (Machine Learning Mastery, 2016).

